# Supplementary material for: An integrated platform for intuitive mathematical programming modeling using LaTeX
Source: PeerJ Comput Sci. 2018 Sep 10;4:e161. doi: 10.7717/peerj-cs.161 (PMC7924498; doi:10.7717/peerj-cs.161)
Supplement: Supplemental Information 1 [file peerj-cs-04-161-s001.pdf]

SUPPLEMENTARY INFORMATION FOR:

An integrated platform for intuitive mathematical programming modeling using LaTeX

Charalampos P. Triantafyllidis[a,b], Lazaros G. Papageorgiou [a,*]

[a] *Centre for Process Systems Engineering, Department of Chemical Engineering, UCL (University College London), UK*
[b] *Smith School of Enterprise and the Environment, University of Oxford, UK*

*Corresponding Author

Email addresses:
h.triantafyllidis@ucl.ac.uk,charalampos.triantafyllidis@smithschool.ox.ac.uk (Charalampos P. Triantafyllidis), l.papageorgiou@ucl.ac.uk (Lazaros G. Papageorgiou)

1. Library of modeling examples

A collection of well-known problems alongside some more executive problems is given, so the user gets a rigid idea of how problems of different formulations and requirements can be represented inside the platform in an admissible manner.

The first two problems, the Transportation problem and the Traveling Salesman problem, are well-known in theoretical computer science with applications that span numerous fields. All the formulations presented below (as raw LaTeX code of course) were the ones also used as input for the platform exactly as shown here.

1.1. The Transportation problem

We wish to find optimum transportation schedule keeping cost of transport minimized. A number of sources distribute a product to a number of destinations and we wish to optimize the effective cost while satisfying the required given demands (Schrijver, 2002). The well-known modeling approach for this problem is given below:

$$
\begin{aligned}
&\texttt{minimize} && \sum_{i,j}(c_{i,j}x_{i,j}) \\
&\texttt{subject to:} && \\
& && \sum_{j}(x_{i,j}) \leq a_i \quad \forall i \\
& && \sum_{i}(x_{i,j}) \geq b_j \quad \forall j \\
& && x \in R_+
\end{aligned}
$$

This problem is the simplest included, as only two sets of constraints are given and the single set of variables belongs to (positive) real numbers. Once we input this problem to the platform with the associated data-set we get the following parser output:

```
        -Reading the variable's types and symbols...
        The optimization problem has :
          (0) binary set(s) of variable(s)
          (0) set(s) of integer variable(s) and
          (1) set(s) of positive real variable(s)
          (0) set(s) of non-negative real variable(s)
        -Reading the objective function definition...
        The optimization sense is: MINIMIZE
        ***Parameter 'c' detected in the constraint***
        **Variable 'x' detected in the objective function with index set: {i,
   j}**
      Found the remaining 1 variables definitions in the constraints!
      -Reading the constraints set (1)...
        ***Parameter 'a' detected in the constraint***
      -Reading the constraints set (2)...
        ***Parameter 'b' detected in the constraint***
Parsing the model terminated successfully... Now printing the Pyomo equivalent
    model...
```

We can see that the parser correctly identified the following:

- the sets and types of variables
- the type of the optimization

- the variables existing in the objective function and the parameters
- an on-the-fly detection of parameters a, b while scanning through the sets of constraints.

The .py model file was generated as follows:

```
########################################
# Define imports of packages:
########################################
from pyomo.environ import *
from pyomo.opt import SolverFactory
model = AbstractModel()
########################################
# Define variables, parameters and sets:
########################################
model.i = Set(dimen=1)
model.j = Set(dimen=1)
########################################
# New parameter detected:
model.c = Param(model.i,model.j, initialize = 0)
########################################
model.x = Var(model.i,model.j, domain=NonNegativeReals)
########################################
# Define objective function:
########################################
def obj_expression(model):
  model.F = sum(model.c[i,j]*model.x[i,j] for i in model.i for j in model.j)
  return model.F
model.OBJ = Objective(rule=obj_expression, sense = minimize)
########################################
# Define constraints:
########################################
########################################
# New parameter detected:
model.a = Param(model.i, initialize = 0)
########################################
def axb_constraint_rule_1(model,i):
  model.C_1= sum(model.x[i,j] for j in model.j) <= model.a[i]
  return model.C_1
model.AxbConstraint_1=Constraint(model.i,rule=axb_constraint_rule_1)
########################################
# New parameter detected:
model.b = Param(model.j, initialize = 0)
########################################
def axb_constraint_rule_2(model,j):
  model.C_2= sum(model.x[i,j] for i in model.i) >= model.b[j]
  return model.C_2
model.AxbConstraint_2=Constraint(model.j,rule=axb_constraint_rule_2)
########################################
# Now link the .data file to the model:
########################################
if __name__ == '__main__':
    opt = SolverFactory('cplex')
    opt.options['threads'] = 12
    opt.options['parallel'] = -1
```

```
    instance = model.create_instance(r'lp_transport.dat')
    results = opt.solve(instance, tee=True)
    results.write()
```

Then, by merging the Pyomo model with the data-set, CPLEX gives:

```
Welcome to IBM(R) ILOG(R) CPLEX(R) Interactive Optimizer 12.6.3.0
  with Simplex, Mixed Integer & Barrier Optimizers
5725-A06 5725-A29 5724-Y48 5724-Y49 5724-Y54 5724-Y55 5655-Y21
Copyright IBM Corp. 1988, 2015.  All Rights Reserved.

Type 'help' for a list of available commands.
Type 'help' followed by a command name for more
information on commands.

CPLEX> Logfile 'cplex.log' closed.
CPLEX> New value for parallel optimization mode: -1
CPLEX> New value for default parallel thread count: 12
Logfile 'cplex.log' open.
Read time = 0.00 sec. (0.00 ticks)
Objective sense      : Minimize
Variables            :       7
Objective nonzeros   :       6
Linear constraints   :       6  [Less: 2,  Greater: 3,  Equal: 1]
  Nonzeros           :      13
  RHS nonzeros       :       6

Variables            : Min LB: 0.0000000        Max UB: all infinite
Objective nonzeros   : Min   : 0.1260000        Max   : 0.2250000
Linear constraints   :
  Nonzeros           : Min   : 1.000000         Max   : 1.000000
  RHS nonzeros       : Min   : 1.000000         Max   : 600.0000
CPLEX> Tried aggregator 1 time.
LP Presolve eliminated 1 rows and 1 columns.
Reduced LP has 5 rows, 6 columns, and 12 nonzeros.
Presolve time = 0.00 sec. (0.00 ticks)

Iteration log . . .
Iteration:     1   Dual objective     =            73.125000

Dual simplex - Optimal:  Objective = 1.5367500000e+002
Solution time =    0.00 sec.  Iterations = 4 (0)
Deterministic time = 0.01 ticks  (10.12 ticks/sec)
```

The associated data-set for this example was :

```
set i := 1 2;
set j := 1 2 3;

param a:=
1  350
2  600;

param b:=
```

```
1    325
2    300
3    275;

param c:=
1 1 0.225
1 2  0.153
1 3  0.162
2 1  0.225
2 2  0.162
2 3  0.126   ;
```

1.2. The Traveling Salesman Problem

One of the most well-known and examined problems, stills being very difficult to solve as the scale of the problem increases is the Traveling Salesman Problem (Applegate et al., 2007), (Johnson, 1990).

$$
\begin{array}{lll}
\texttt{minimize} & \sum_{i,j:i\neq j} (c_{i,j} x_{i,j}) & \\
\texttt{subject to:} & & \\
 & \sum_{j:i\neq j} (x_{i,j}) = 1 & \forall i \\
 & \sum_{i:i\neq j} (x_{i,j}) = 1 & \forall j \\
 & u_i - u_j + n * x_{i,j} \leq n-1 & \forall i \geq 2, i \neq j, j \leq n \\
 & u \in Z_+, x \in \{0,1\} &
\end{array}
$$

The parser correctly converts the model and the output is given below:

```
        -Reading the variable's types and symbols...
        The optimization problem has :
          (1) binary set(s) of variable(s)
          (0) set(s) of integer variable(s) and
          (0) set(s) of positive real variable(s)
          (0) set(s) of non-negative real variable(s)
          (1) set(s) of positive integer variable(s)
        -Reading the objective function definition...
        The optimization sense is: MINIMIZE
        ***Parameter 'c' detected in the constraint***
        **Variable 'x' detected in the objective function with index set: {i,
   j}**
        1 out of total 2 sets of variables were not detected in the objective
    function...
        Searching for the rest of the variables in the model...
            Found set(s) of variable(s) u with index set {i}
    Found the remaining 1 variables definitions in the constraints!
    -Reading the constraints set (1)...
    -Reading the constraints set (2)...
    -Reading the constraints set (3)...
        ***Parameter 'n' detected in the constraint***
Parsing the model terminated successfully... Now printing the Pyomo equivalent
    model...
```

The produced Pyomo model is as follows:

```
##########################################
# Define imports of packages:
##########################################
from pyomo.environ import *
from pyomo.opt import SolverFactory
model = AbstractModel()
##########################################
# Define variables, parameters and sets:
##########################################
model.i = Set(dimen=1)
model.j = Set(dimen=1)
##########################################
# New parameter detected:
model.c = Param(model.i,model.j, initialize = 0)
##########################################
model.x = Var(model.i,model.j, domain=Binary)
model.u = Var(model.i, domain=PositiveIntegers)
##########################################
# Define objective function:
##########################################
def obj_expression(model):
  model.F = sum(model.c[i,j]*model.x[i,j] for i in model.i for j in model.j if i!=j)
  return model.F
model.OBJ = Objective(rule=obj_expression, sense = minimize)
##########################################
# Define constraints:
##########################################
def axb_constraint_rule_1(model,i):
  model.C_1= sum(model.x[i,j] for j in model.j if i!=j) == 1
  return model.C_1
model.AxbConstraint_1=Constraint(model.i,rule=axb_constraint_rule_1)
def axb_constraint_rule_2(model,j):
  model.C_2= sum(model.x[i,j] for i in model.i if i!=j) == 1
  return model.C_2
model.AxbConstraint_2=Constraint(model.j,rule=axb_constraint_rule_2)
##########################################
# New parameter detected:
model.n = Param(initialize = 0)
##########################################
def axb_constraint_rule_3(model,i,j):
  if i>=2 and i!=j and j<=model.n:
      model.C_3= model.u[i]-model.u[j]+model.n*model.x[i,j] <= model.n-1
      return model.C_3
  else:
      return Constraint.Skip
model.AxbConstraint_3=Constraint(model.i,model.j,rule=axb_constraint_rule_3)
##########################################
# Now link the .data file to the model:
##########################################
if __name__ == '__main__':
    opt = SolverFactory('cplex')
```

```
    opt.options['threads'] = 12
    opt.options['parallel'] = -1
    instance = model.create_instance(r'TSP.dat')
    results = opt.solve(instance, tee=True)
    results.write()
```

Solving the model with the associated data-set gives:

```
Welcome to IBM(R) ILOG(R) CPLEX(R) Interactive Optimizer 12.6.3.0
  with Simplex, Mixed Integer & Barrier Optimizers
5725-A06 5725-A29 5724-Y48 5724-Y49 5724-Y54 5724-Y55 5655-Y21
Copyright IBM Corp. 1988, 2015.  All Rights Reserved.

Type 'help' for a list of available commands.
Type 'help' followed by a command name for more
information on commands.

CPLEX> Logfile 'cplex.log' closed.
CPLEX> New value for parallel optimization mode: -1
Logfile 'cplex.log' open.
CPLEX> New value for default parallel thread count: 12
Read time = 0.00 sec. (0.00 ticks)
Objective sense      : Minimize
Variables            :      17  [Nneg: 1,  Binary: 12,  General Integer: 4]
Objective nonzeros   :      12
Linear constraints   :      18  [Less: 9,  Equal: 9]
  Nonzeros           :      52
  RHS nonzeros       :      18

Variables            : Min LB: 0.0000000        Max UB: 1.000000
Objective nonzeros   : Min   : 2.000000         Max   : 100.0000
Linear constraints   :
  Nonzeros           : Min   : 1.000000         Max   : 4.000000
  RHS nonzeros       : Min   : 1.000000         Max   : 3.000000
CPLEX> Tried aggregator 1 time.
MIP Presolve eliminated 4 rows and 2 columns.
Reduced MIP has 14 rows, 15 columns, and 42 nonzeros.
Reduced MIP has 12 binaries, 3 generals, 0 SOSs, and 0 indicators.
Presolve time = 0.00 sec. (0.02 ticks)
Probing time = 0.00 sec. (0.01 ticks)
Tried aggregator 1 time.
Reduced MIP has 14 rows, 15 columns, and 42 nonzeros.
Reduced MIP has 12 binaries, 3 generals, 0 SOSs, and 0 indicators.
Presolve time = 0.00 sec. (0.02 ticks)
Probing time = 0.00 sec. (0.01 ticks)
Clique table members: 11.
MIP emphasis: balance optimality and feasibility.
MIP search method: dynamic search.
Parallel mode: opportunistic, using up to 12 threads.
Root relaxation solution time = 0.00 sec. (0.02 ticks)

        Nodes                                          Cuts/
   Node  Left     Objective  IInf  Best Integer     Best Bound    ItCnt     Gap

```

```
*      0+     0                              302.0000         0.0000
    100.00%
*      0      0       integral      0        14.0000        14.0000          6
    0.00%
Elapsed time = 0.00 sec. (0.19 ticks, tree = 0.00 MB, solutions = 2)

Root node processing (before b&c):
  Real time             =    0.00 sec. (0.19 ticks)
Parallel b&c, 12 threads:
  Real time             =    0.00 sec. (0.00 ticks)
  Sync time (average)   =    0.00 sec.
  Wait time (average)   =    0.00 sec.
                          ------------
Total (root+branch&cut) =    0.00 sec. (0.19 ticks)

Solution pool: 2 solutions saved.

MIP - Integer optimal solution:  Objective = 1.4000000000e+001
Solution time =    0.00 sec.  Iterations = 6  Nodes = 0
Deterministic time = 0.19 ticks  (192.41 ticks/sec)
```

The data-set for this demonstration was:

```
set i := 1 2 3 4;
set j := 1 2 3 4;
param n:= 4 ;
param c:=
    1 1  100
    1 2  2
    1 3  100
    1 4  100
    2 1  100
    2 2  100
    2 3  3
    2 4  100
    3 1  100
    3 2  100
    3 3  100
    3 4  4
    4 1  5
    4 2  100
    4 3  100
    4 4  100  ;
```

1.3. Cancer Therapy

The model which was original published in (Knijnenburg et al., 2016) and attempts to binarize continuous outputs for cancer research, via Binary-Integer Optimization, based on a Boolean-Function Synthesis formulation:

$$
\begin{array}{lll}
\texttt{minimize} & -\sum_{n:y_n=0}(w_n y2_n) - \sum_{n:y_n=1}(w_n y2_n) & \\
\texttt{subject to:} & & \\
 & sa_{p,k} + sb_{p,k} \leq 1 & \forall p,k \\
 & \sum_p (sa_{p,k} + sb_{p,k}) \leq M & \forall k \\
 & g * t_{n,k} \leq \sum_{p:X_{n,p}=1}(1 - sb_{p,k}) + \sum_{p:X_{n,p}=0}(1 - sa_{p,k}) & \forall n,k \\
 & \sum_{p:X_{n,p}=1}(1 - sb_{p,k}) + \sum_{p:X_{n,p}=0}(1 - sa_{p,k}) \leq t_{n,k} + g - 1 & \forall n,k \\
 & y2_n \leq \sum_k (t_{n,k}) & \forall n \\
 & \sum_k (t_{n,k}) \leq d * y2_n & \forall n \\
 & t, sa, sb, y2 \in \{0,1\} &
\end{array}
$$

The parser gives:

```
        -Reading the variable's types and symbols...
        The optimization problem has :
          (4) binary set(s) of variable(s)
          (0) set(s) of integer variable(s) and
          (0) set(s) of positive real variable(s)
          (0) set(s) of non-negative real variable(s)
        -Reading the objective function definition...
        The optimization sense is: MINIMIZE
        ***Parameter 'w' detected in the constraint***
        **Variable 'y2' detected in the objective function with index set: {n}**
        3 out of total 4 sets of variables were not detected in the objective function...
        Searching for the rest of the variables in the model...
            Found set(s) of variable(s) sa with index set {p,k}
            Found set(s) of variable(s) sb with index set {p,k}
            Found set(s) of variable(s) t with index set {n,k}
    Found the remaining 3 variables definitions in the constraints!
    -Reading the constraints set (1)...
    -Reading the constraints set (2)...
        ***Parameter 'M' detected in the constraint***
    -Reading the constraints set (3)...
        ***Parameter 'g' detected in the constraint***
    -Reading the constraints set (4)...
    -Reading the constraints set (5)...
    -Reading the constraints set (6)...
        ***Parameter 'd' detected in the constraint***
Parsing the model terminated successfully... Now printing the Pyomo equivalent model...
```

The Pyomo output of the parser is:

```
######################################
# Define imports of packages:
######################################
```

```
from pyomo.environ import *
from pyomo.opt import SolverFactory
model = AbstractModel()
########################################
# Define variables, parameters and sets:
########################################
model.n = Set(dimen=1)
########################################
# New parameter detected:
model.w = Param(model.n, initialize = 0)
########################################
model.y2 = Var(model.n, domain=Binary)
#########################################
# New indexing parameter detected...
model.y = Param(model.n, initialize = 0)
#########################################
model.p = Set(dimen=1)
model.k = Set(dimen=1)
model.sa = Var(model.p,model.k, domain=Binary)
model.sb = Var(model.p,model.k, domain=Binary)
model.t = Var(model.n,model.k, domain=Binary)
########################################
# Define objective function:
########################################
def obj_expression(model):
  model.F = sum(-model.w[n]*model.y2[n] for n in model.n if model.y[n]==0)-sum
    (model.w[n]*model.y2[n] for n in model.n if model.y[n]==1)
  return model.F
model.OBJ = Objective(rule=obj_expression, sense = minimize)
########################################
# Define constraints:
########################################
def axb_constraint_rule_1(model,p,k):
  model.C_1= model.sa[p,k]+model.sb[p,k] <= 1
  return model.C_1
model.AxbConstraint_1=Constraint(model.p,model.k,rule=axb_constraint_rule_1)
########################################
# New parameter detected:
model.M = Param(initialize = 0)
########################################
def axb_constraint_rule_2(model,k):
  model.C_2= sum(model.sa[p,k]+model.sb[p,k] for p in model.p) <= model.M
  return model.C_2
model.AxbConstraint_2=Constraint(model.k,rule=axb_constraint_rule_2)
########################################
# New parameter detected:
model.g = Param(initialize = 0)
########################################
#########################################
# New indexing parameter detected...
model.X = Param(model.n,model.p, initialize = 0)
#########################################
def axb_constraint_rule_3(model,n,k):
  model.C_3= model.g*model.t[n,k] <= sum(1-model.sb[p,k] for p in model.p if
```

```
    model.X[n,p]==1)+sum(1-model.sa[p,k] for p in model.p if model.X[n,p]==0)
  return model.C_3
model.AxbConstraint_3=Constraint(model.n,model.k,rule=axb_constraint_rule_3)
def axb_constraint_rule_4(model,n,k):
  model.C_4= sum(1-model.sb[p,k] for p in model.p if model.X[n,p]==1)+sum(1-
    model.sa[p,k] for p in model.p if model.X[n,p]==0) <= model.t[n,k]+model.g
    -1
  return model.C_4
model.AxbConstraint_4=Constraint(model.n,model.k,rule=axb_constraint_rule_4)
def axb_constraint_rule_5(model,n):
  model.C_5= model.y2[n] <= sum(model.t[n,k] for k in model.k)
  return model.C_5
model.AxbConstraint_5=Constraint(model.n,rule=axb_constraint_rule_5)
##########################################
# New parameter detected:
model.d = Param(initialize = 0)
##########################################
def axb_constraint_rule_6(model,n):
  model.C_6= sum(model.t[n,k] for k in model.k) <= model.d*model.y2[n]
  return model.C_6
model.AxbConstraint_6=Constraint(model.n,rule=axb_constraint_rule_6)
##########################################
# Now link the .data file to the model:
##########################################
if __name__ == '__main__':
    opt = SolverFactory('cplex')
    opt.options['threads'] = 12
    opt.options['parallel'] = -1
    instance = model.create_instance(r'LOBICO.dat')
    results = opt.solve(instance, tee=True)
    results.write()
```

CPLEX converged as follows:

```
Welcome to IBM(R) ILOG(R) CPLEX(R) Interactive Optimizer 12.6.3.0
  with Simplex, Mixed Integer & Barrier Optimizers
5725-A06 5725-A29 5724-Y48 5724-Y49 5724-Y54 5724-Y55 5655-Y21
Copyright IBM Corp. 1988, 2015.  All Rights Reserved.

Type 'help' for a list of available commands.
Type 'help' followed by a command name for more
information on commands.

CPLEX> Logfile 'cplex.log' closed.
CPLEX> New value for parallel optimization mode: -1
CPLEX> New value for default parallel thread count: 12
Logfile 'cplex.log' open.
Read time = 0.03 sec. (2.36 ticks)
Objective sense      : Minimize
Variables            :    2167  [Nneg: 1,  Binary: 2166]
Objective nonzeros   :     642
Linear constraints   :    3975  [Less: 3974,  Equal: 1]
  Nonzeros           :  160981
  RHS nonzeros       :    2691
```

```
Variables              : Min LB: 0.0000000        Max UB: 1.000000
Objective nonzeros     : Min   : 1.000000e-005    Max   : 0.03049700
Linear constraints     :
  Nonzeros             : Min   : 1.000000         Max   : 60.00000
  RHS nonzeros         : Min   : 1.000000         Max   : 60.00000
CPLEX> Tried aggregator 1 time.
MIP Presolve eliminated 1958 rows and 3 columns.
MIP Presolve added 1106 rows and 0 columns.
MIP Presolve modified 78324 coefficients.
Reduced MIP has 3123 rows, 2164 columns, and 82967 nonzeros.
Reduced MIP has 2164 binaries, 0 generals, 0 SOSs, and 0 indicators.
Presolve time = 0.19 sec. (348.06 ticks)
Found incumbent of value 0.000008 after 0.25 sec. (471.51 ticks)
Probing time = 0.00 sec. (7.15 ticks)
Tried aggregator 1 time.
Reduced MIP has 3123 rows, 2164 columns, and 82967 nonzeros.
Reduced MIP has 2164 binaries, 0 generals, 0 SOSs, and 0 indicators.
Presolve time = 0.05 sec. (106.61 ticks)
Probing time = 0.02 sec. (7.22 ticks)
Clique table members: 4098.
MIP emphasis: balance optimality and feasibility.
MIP search method: dynamic search.
Parallel mode: opportunistic, using up to 12 threads.
Root relaxation solution time = 0.28 sec. (345.09 ticks)

        Nodes                                         Cuts/
   Node  Left     Objective  IInf  Best Integer    Best Bound    ItCnt     Gap

*     0+    0                          0.0000        -1.0000               ---
      0     0       -0.5507  1644      0.0000        -0.5507     1549      ---
*     0+    0                         -0.1103        -0.5507
   399.45%
*     0+    0                         -0.2646        -0.5507
   108.13%
      0     0       -0.5442  1608     -0.2646     Cuts: 133     1601
   105.68%
      0     0       -0.5410  1605     -0.2646     Cuts: 206     1631
   104.46%
      0     0       -0.5101  1843     -0.2646      Cuts: 23     1857
   92.78%
      0     0        cutoff           -0.2646        -0.2646     1915
   0.00%
Elapsed time = 2.28 sec. (2770.31 ticks, tree = 0.01 MB, solutions = 3)

Zero-half cuts applied:  262
Lift and project cuts applied:  2
Gomory fractional cuts applied:  7

Root node processing (before b&c):
  Real time             =    2.28 sec. (2770.42 ticks)
Parallel b&c, 12 threads:
  Real time             =    0.00 sec. (0.00 ticks)
  Sync time (average)   =    0.00 sec.
```

```
  Wait time (average)    =     0.00 sec.
                          ------------
Total (root+branch&cut) =     2.28 sec. (2770.42 ticks)

Solution pool: 3 solutions saved.

MIP - Integer optimal solution:  Objective = -2.6459700000e-001
Solution time =     2.28 sec.  Iterations = 1915  Nodes = 0
Deterministic time = 2770.43 ticks  (1214.57 ticks/sec)
```

The data file can be found online using the manuscript given links, and as being very large it was omitted here. The value of the objective function for the supplied example was verified to be correct by comparing it with the equivalent MATLAB model that the authors developed and distributed.

2. Other modeling examples

Examples of MILP and MIQP formulations which were successfully parsed by the platform are given below.

2.1. Optimal Process Plant Layout

Designing the layout of a chemical plant involves decisions concerning the spatial allocation of equipment items and the required connections among them. This problem is known as the *layout problem* and the model we used below was originally presented in (Papageorgiou and Rotstein, 1998):

$$
\begin{array}{lll}
\texttt{minimize} & \sum_{i,j:i\neq j} (C_{i,j} D_{i,j}) & \\
\texttt{subject to:} & & \\
 & D_{i,j} = R_{i,j} + LF_{i,j} + A_{i,j} + B_{i,j} & \forall i < |i|, j \geq i+1 \\
 & l_i = a_i o_i + b_i (1 - o_i) & \forall i \\
 & d_i = a_i + b_i - l_i & \forall i \\
 & x_i \geq \frac{l_i}{2} & \forall i \\
 & y_i \geq \frac{d_i}{2} & \forall i \\
 & R_{i,j} - LF_{i,j} = x_i - x_j & \forall i < |i|, j \geq i+1 \\
 & A_{i,j} - B_{i,j} = y_i - y_j & \forall i < |i|, j \geq i+1 \\
 & x_i - x_j + M(E1_{i,j} + E2_{i,j}) \geq \frac{l_i}{2} + \frac{l_j}{2} & \forall i < |i|, j \geq i+1 \\
 & x_j - x_i + M(1 - E1_{i,j} + E2_{i,j}) \geq \frac{l_i}{2} + \frac{l_j}{2} & \forall i < |i|, j \geq i+1 \\
 & y_i - y_j + M(1 + E1_{i,j} - E2_{i,j}) \geq \frac{d_i}{2} + \frac{d_j}{2} & \forall i < |i|, j \geq i+1 \\
 & y_j - y_i + M(2 - E1_{i,j} - E2_{i,j}) \geq \frac{d_i}{2} + \frac{d_j}{2} & \forall i < |i|, j \geq i+1 \\
 & l, LF, d, x, y, A, B, D, R \in R_+, o, E1, E2 \in \{0, 1\} &
\end{array}
$$

The parser output is the following one:

```
        -Reading the variable's types and symbols...
        The optimization problem has :
          (3) binary set(s) of variable(s)
          (0) set(s) of integer variable(s) and
          (9) set(s) of positive real variable(s)
          (0) set(s) of non-negative real variable(s)
        -Reading the objective function definition...
        The optimization sense is: MINIMIZE
        ***Parameter 'C' detected in the constraint***
        **Variable 'D' detected in the objective function with index set: {i,
j}**
        11 out of total 12 sets of variables were not detected in the
objective function...
        Searching for the rest of the variables in the model...
            Found set(s) of variable(s) LF with index set {i,j}
            Found set(s) of variable(s) A with index set {i,j}
            Found set(s) of variable(s) R with index set {i,j}
            Found set(s) of variable(s) l with index set {i}
            Found set(s) of variable(s) o with index set {i}
            Found set(s) of variable(s) d with index set {i}
            Found set(s) of variable(s) x with index set {i}
```

```
            Found set(s) of variable(s) y with index set {i}
            Found set(s) of variable(s) B with index set {i,j}
            Found set(s) of variable(s) E1 with index set {i,j}
            Found set(s) of variable(s) E2 with index set {i,j}
    Found the remaining 11 variables definitions in the constraints!
    -Reading the constraints set (1)...
    -Reading the constraints set (2)...
        ***Parameter 'a' detected in the constraint***
        ***Parameter 'b' detected in the constraint***
    -Reading the constraints set (3)...
    -Reading the constraints set (4)...
    -Reading the constraints set (5)...
    -Reading the constraints set (6)...
    -Reading the constraints set (7)...
    -Reading the constraints set (8)...
        ***Parameter 'M' detected in the constraint***
    -Reading the constraints set (9)...
    -Reading the constraints set (10)...
    -Reading the constraints set (11)...
Parsing the model terminated successfully... Now printing the Pyomo equivalent
    model...
```

2.2. Community detection via modularity metric optimization

The detection of community structure has been used to reveal the relationships between individual objects and their groupings in networks. A mathematical programming approach to identify the optimal community structures in complex networks based on the maximization of a network modularity metric for partitioning a network into modules is formulated as a mixed integer quadratic programming (MIQP) model. This problem is known as *Modularity Optimization* and the model we used below was originally presented in (Xu et al., 2007):

$$
\begin{aligned}
&\texttt{maximize} && \sum_m \left(\frac{L_m}{Lambda} - \left(\frac{D_m}{2Lambda}\right)^2\right) \\
&\texttt{subject to:} && \\
& && \sum_m (Y_{n,m}) = 1 && \forall n \\
& && X_{n,e,m} \le Y_{n,m} && \forall m, (n,e) \in LK \\
& && X_{n,e,m} \le Y_{e,m} && \forall m, (n,e) \in LK \\
& && L_m = \sum_{n,e:(n,e)\in LK} (X_{n,e,m}) && \forall m \\
& && D_m = \sum_n (delta_n Y_{n,m}) && \forall m \\
& && E_m \le E_{m-1} \quad \forall m > 1 && \\
& && \sum_{n,e:(n,e)\in LK} (X_{n,e,m}) \ge a * E_m && \forall m \\
& && \sum_{n,e:(n,e)\in LK} (X_{n,e,m}) \le b * E_m && \forall m \\
& && L_m - L_k \le epsilon + b(1 - E_k) && \forall m, k > m \\
& && L_k - L_m \le epsilon + b(1 - E_k) && \forall m, k > m \\
& && L, D \in R_+, E, X, Y \in \{0,1\} &&
\end{aligned}
$$

The parser has to identify on-the-fly an array of parameters as shown below:

```
        -Reading the variable's types and symbols...
        The optimization problem has :
          (3) binary set(s) of variable(s)
          (0) set(s) of integer variable(s) and
          (2) set(s) of positive real variable(s)
          (0) set(s) of non-negative real variable(s)
        -Reading the objective function definition...
        The optimization sense is: MAXIMIZE
        ------------------------------------------------
        Quadratic Programming Objective function detected!!!
        ------------------------------------------------
        ***Parameter 'Lambda' detected in the constraint***
        **Variable 'L' detected in the objective function with index set: {m
    }**
        **Variable 'D' detected in the objective function with index set: {m
    }**
        3 out of total 5 sets of variables were not detected in the objective
     function...
        Searching for the rest of the variables in the model...
            Found set(s) of variable(s) Y with index set {n,m}
            Found set(s) of variable(s) X with index set {n,e,m}
            Found set(s) of variable(s) E with index set {m}
     Found the remaining 3 variables definitions in the constraints!
```

```
    -Reading the constraints set (1)...
    -Reading the constraints set (2)...
    -Reading the constraints set (3)...
    -Reading the constraints set (4)...
    -Reading the constraints set (5)...
        ***Parameter 'delta' detected in the constraint***
    -Reading the constraints set (6)...
    -Reading the constraints set (7)...
        ***Parameter 'a' detected in the constraint***
    -Reading the constraints set (8)...
        ***Parameter 'b' detected in the constraint***
    -Reading the constraints set (9)...
        ***Parameter 'epsilon' detected in the constraint***
    -Reading the constraints set (10)...
Parsing the model terminated successfully... Now printing the Pyomo equivalent
    model...
```

Model name / Step	Conversion from LaTeX	Pyomo model generation	Solver (CPLEX)
LP Transportation problem	0.048	0.034	0.00
Traveling Salesman Problem (TSP)	0.049	0.026	0.02
Cancel Therapy	0.060	4.520	4.00
Optimal Process Plant Layout	0.109	0.151	1.14
Community Detection via Modularity	0.054	0.192	0.86

Table 1: Cpu-time (in seconds) for the modeling steps. The computational environment used was: Intel Core i5 8250U @1.6Ghz , 8GB RAM 2.4 Ghz, Windows 10 x64, Python 3.6

References

Applegate, D.L., Bixby, R.E., Chvatal, V., Cook, W.J., 2007. The Traveling Salesman Problem: A Computational Study (Princeton Series in Applied Mathematics). Princeton University Press, Princeton, NJ, USA.

Johnson, D.S., 1990. Local optimization and the Traveling Salesman Problem. Springer Berlin Heidelberg, Berlin, Heidelberg. pp. 446–461.

Knijnenburg, T.A., Klau, G.W., Iorio, F., Garnett, M.J., McDermott, U., Shmulevich, I., Wessels, L.F.A., 2016. Logic models to predict continuous outputs based on binary inputs with an application to personalized cancer therapy. Scientific Reports 6, 36812. URL: http://dx.doi.org/10.1038/srep36812, doi:10.1038/srep36812.

Papageorgiou, L.G., Rotstein, G.E., 1998. Continuous-domain mathematical models for optimal process plant layout. Industrial & Engineering Chemistry Research 37, 3631–3639. URL: http://dx.doi.org/10.1021/ie980146v, doi:10.1021/ie980146v, arXiv:http://dx.doi.org/10.1021/ie980146v.

Schrijver, A., 2002. On the history of the transportation and maximum flow problems. Mathematical Programming 91, 437–445.

Xu, G., Tsoka, S., Papageorgiou, L.G., 2007. Finding community structures in complex networks using mixed integer optimisation. The European Physical Journal B 60, 231–239. URL: https://doi.org/10.1140/epjb/e2007-00331-0, doi:10.1140/epjb/e2007-00331-0.
